# Traditional Sensory Evaluation and Bionic Electronic Nose as Innovative Tools for the Packaging Performance Evaluation of Chitosan Film

**DOI:** 10.3390/polym12102310

**Published:** 2020-10-09

**Authors:** Wei Song, Jian Xu, Lili Ren, Li Guo, Jin Tong, Liyan Wang, Zhiyong Chang

**Affiliations:** 1 Key Laboratory of Bionic Engineering (Ministry of Education), College of Biological and Agricultural Engineering, Jilin University, Changchun 130022, China; songwei20@mails.jlu.edu.cn (W.S.); xujian19@mails.jlu.edu.cn (J.X.); liguo2012@jlu.edu.cn (L.G.); jtong@jlu.edu.cn (J.T.); wangliyan@jlau.edu.cn (L.W.); 2 College of Food Science and Engineering, Jilin Agricultural University, Changchun 130118, China

**Keywords:** packaging film, chitosan film, sensory evaluation, electronic nose

## Abstract

Inspired by the natural epidermis of animals and plants with antioxidant and antibacterial properties, the aim of this research was to characterize and analyze the effects of the chitosan concentrations on properties of glycerol plasticized chitosan (GPC) film and to investigate the suitability of sensory evaluation and bionic electronic nose (b-electronic nose) detection to assess the freshness of ground beef packaged in the GPC film. The increase in chitosan concentration resulted in an increase in solubility value, total color differences and color intensity of chitosan films. The water vapor permeability (WVP) of the GPC films decreased with the increasing of the chitosan concentration and then increased at higher chitosan concentrations. Longer storage time led to poorer freshness of the ground beef and the GPC film could keep beef samples fresher and delay the deterioration of the beef. Both the traditional sensory evaluation and b-electronic nose technology were fit for evaluating the quality and shelf-life of ground beef, which could advantageously be applied in the future for analyzing other bionic food packaging materials.

## 1. Introduction

The modern food packaging industry is facing two major challenges. The growing accumulation of petrochemical-based plastic packaging waste together with the difficulty of recycling the majority of packaging products has increased the interest in biodegradable packaging materials from renewable and natural sources. The continuously increasing interest of consumers in better fresh-like quality, longer shelf-life, fewer preservatives, convenience and safety of food has encouraged research into active packaging. Active packaging is defined as a system that can extend shelf-life and improve safety or sensory properties while maintaining the quality of the food though incorporating active compounds and ingredients into packaging materials [1,2]. Active food packaging materials based on biodegradable and natural polymers, such as polysaccharides, proteins and natural gums, have been widely investigated because they can be a viable solution to the waste disposal of plastic packaging and can also improve food quality and extend food’s shelf-life. Active edible film or coating with antimicrobial properties can be considered as one of the most promising active packaging systems, which acts as a barrier to the external elements, such as moisture, oxygen, oil and microorganisms, to improve the quality of food products [3,4].

In nature, many animal and plant epidermis with antioxidant and antimicrobial properties were regarded as active packaging materials, such as crab and crawfish shell [5,6,7,8,9], fish skins [10], apple tree bark [11] and mangosteen pericarp [12]. The main ingredient of shells of crustaceans, such as crab, shrimp and crawfish, is chitin, which is the second most abundant polysaccharide found in nature after cellulose [5,6,7,8,9]. One of the most promising active edible films is the one based on chitosan, a linear polysaccharide consisting of (1,4)-linked 2-amino-deoxy-*β*-D-glucan, obtained by deacetylation of chitin. Due to its non-toxicity, biodegradability, biocompatibility, strong antimicrobial and antifungal activities and film forming property, chitosan-based films bring some advantages over other biomolecule-based ones used as packaging materials for the quality preservation of a variety of foods [8,13,14,15]. Edible films and coatings based on chitosan combined with different materials such as plant and animal proteins [16,17,18] as well as polysaccharides [19,20,21] have attracted serious attention in food preservation and packaging technology. These studies showed that incorporation of chitosan significantly improved the mechanical properties, water vapor transport and gas permeation ability of the blend films, and chitosan exhibited high antimicrobial activity against pathogenic and spoilage micro-organisms. Due to its unique cationic character, chitosan can bind to many different food components through donor/acceptor interaction. However, the antimicrobial activity of chitosan could be decreased in the above-mentioned complex food matrices [8], which is a good indication that real food systems are complex matrices and the promising results obtained in vitro with model systems in buffers or microbiological media are not necessarily applicable to the real ones [8].

The plasticizer agents play an important role in improving the flexibility [22], mechanical and barrier properties [23], and chain mobility [24], of bio-based polymer films. Glycerol, a major by-product in biodiesel production and one of the most important natural plasticizers, is commonly recognized as one of the most suitable plasticizer agents in the bio-polymer film-preparation techniques due to its stability and compatibility with hydrophilic matrix [25,26], which increases the value of glycerol from a low-grade by-product to a useful additive. Glycerol is widely used as a plasticizer to overcome the brittleness of starches to develop thermoplastic starch by reducing intra and intermolecular hydrogen bonds [27]. Glycerol used in the chitosan films can also increase the intramolecular distance and the hydrophilic character of glycerol can improve the adsorption and desorption of moisture of the chitosan films [28]. Meanwhile, with the increase in the glycerol concentration, the elongation at break of chitosan films increased and tensile strength decreased because the small sized glycerol molecules could penetrate the polymer chain, leading to weakening of the inter-chain interactions [28,29]. It is evident that the addition of glycerol plasticizer will also influence other properties of the bio-polymer films.

The quality and shelf-life of meat are frequently assessed by performing sensory analyses, monitoring changes in microbial levels over time and monitoring changes in some of the chemical metabolites produced during meat spoilage by using an electronic nose system [30,31,32]. Sensory evaluation is a subjective method that can estimate the sensory quality, including olfactory sensations, taste, flavor, and texture of food. Electronic nose, as a detection system that simulates biological olfactory patterns, is a device with many advantages over other instrumental methods of food freshness assessment, such as low cost, high speed, simplicity, high sensitivity, and a small amount of sample. However, the suitability of sensory and bionic electronic nose (b-electronic nose) methods to evaluate the effect of chitosan edible films on the quality and shelf-life of ground beef was not taken into consideration in the literature. Therefore, the present study aims to prepare and characterize glycerol plasticized chitosan (GPC) films, analyzing the influence of the chitosan concentration on physicochemical, mechanical and barrier properties and then to test the potential of investigating the freshness of ground beef sealed with GPC films by means of traditional sensory analysis and the b-electronic nose approach, which are relevant for GPC films used as active packaging materials in real food preservation.

## 2. Materials and Methods

### 2.1. Materials

Chitosan with 88.0% deacetylation degree and a molecular weight of 60 kDa was obtained from Sinopharm Chemical Reagent Co. Ltd. No.20120330 (Shanghai, China). The ground beef samples containing 20% fat and 80% lean and polyethylene films were purchased from the Ouya Supermarket (Changchun, China). Glycerol, acetic acid (36%), anhydrous CaCl_2_ and KNO_3_ were obtained from Beijing Beihua Fine Chemicals Co. Ltd. (Beijing, China). All these materials were used without further purification.

### 2.2. GPC Film Preparation

Chitosan powder (1, 2, 3 and 4 (g)) was dissolved in 100 mL of 0.2 M acetic acid aqueous solution at 60 °C with stirring of 600 rpm to obtain chitosan solution. Glycerol was added to the chitosan solution as a plasticizer at a concentration of 20 wt% of the chitosan powder. After the mixture was stirred with 600 rpm for 30 min at 60 °C and degassed, 40 (g) of chitosan solution was distributed into Petri dishes with the diameter of 75 mm by casting and dried at 40 °C and 30% relative humidity for 30 h. The peeled chitosan films were kept at room temperature and 75% relative humidity (RH) for 48 h prior to the determination of their physicochemical, barrier and mechanical properties. The water contents of the chitosan films after being conditioned in 75% RH to moisture equilibrium were 30.18 ± 0.12, 32.73 ± 0.10, 35.99 ± 0.23 and 35.83 ± 0.27 for chitosan concentrations of 1%, 2%, 3% and 4%, respectively. In addition, the thicknesses of the films for chitosan concentrations of 1%, 2%, 3% and 4% were 0.12 ± 0.01, 0.19 ± 0.04, 0.20 ± 0.03 and 0.21 ± 0.05, respectively.

### 2.3. Properties of GPC Films

Chitosan films were characterized with Fourier transform infrared (FT-IR) Spectrometer (IRAffinity-1 spectrophotometer, Shimadzu, Japan) to investigate the interactions in the films. A spectral resolution of 4 cm^−1^ was employed and 32 scans were acquired. Attenuated total reflectance-Fourier transform infrared analysis (ATR-FTIR) spectra of the chitosan film were measured according to Ren, et al., 2017 [23].

X-ray diffraction (XRD) pattern of the GPC film was measured by using a Rigaku D/max-2500 X-ray diffractometer (Rigaku Corporation, Tokyo, Japan) with Cu-Ka radiation (λ = 1.542 Å) at 40 kV and 250 mA. The XRD patterns were obtained at 25 °C over the 2θ range of 3–35° at a speed of 2°/min.

The density of the GPC film was determined from the specimen weight and volume. Solubility (WS) and swelling degree (SD) of the GPC film in water were measured according to the method described in the literature with minor modification [33,34]. The chitosan films were cut into 20 mm × 20 mm strips and stored in a desiccator with 0% RH to keep their weight constant. Then, the strips were weighed and placed in glass vessels with 40 mL of deionized water. After that, the strips were maintained with constant agitation at 80 ℃ for 2 h, the remaining water was discarded, and then the strips were superficially dried with filter paper and weighed to obtain the wet weight. Finally, the strips were dried at 105 ℃ until a constant weight was obtained. WS and SD of the chitosan film were calculated as follows:

(1) $$WS\;\left(\%\right)\;=\;\frac{Initial\;dry\;weight\;-\;Final\;dry\;weight}{Initial\;dry\;weight}\times 100$$

(2) $$SD\;\left(\%\right)\;=\;\frac{Wet\;weight\;-\;Initial\;dry\;weight}{Initial\;dry\;weight}\times 100$$

The color, water vapor permeability (WVP) and mechanical properties of the GPC film were determined according to the method described by Ren et al. [35]. At least five specimens were measured for each experimental condition and the average values were taken.

The tensile fractured surface morphology of GPC films was studied by using a scanning electron microscope (Zeiss EVO 18 SEM, Oberkochen, Germany). The samples were coated with a thin gold layer with the help of gold sputter and then observed and photographed. An accelerating potential of 20 kV was used during tests.

### 2.4. Sensory Evaluation

Sensory evaluation (scoring test) was assessed according to the sensory index of Chinese meat hygiene standard (GB 2707). Twenty experienced assessors (M = 10, F = 10, mean age = 25, age range from 20 to 38) were recruited among staff and students of Jilin University (Changchun, China). First, they were informed of the purpose and background of this study, and then were trained for two weeks to learn the color, odor, viscosity, and resilience evaluation methods and terminology. Thirdly, they were trained to observe and evaluate known samples provided by the author and then the unknown samples for this study. Beef sample (5 g) was put into a 50 mL glass bottle, sealed with the chitosan film (2% chitosan concentration) or polyethylene film and then conditioned at 8 ℃ and 70% RH prior to the determinations of the freshness of beef evaluated by sensory evaluation.

Sensory evaluation was assessed using indicators such as color (muscle gloss, interstitial fluid color), odor (meat specific odor, putrid smell), viscosity (the surface viscosity and the interstitial fluid amount of the latest slice was felt) and resilience (the recovery rate of a sunk part after pressing with the fingers). After the evaluation, the assessors were asked to divide the samples into three groups, including the fresh group, sub-fresh group and putrid group. Scoring tests were conducted to quantify the freshness of the beef samples using a 5-point scale (5 = best, 3 = medium, 1 = worst). If the score is less than 2, it is considered that the sample is unacceptable to consumers.

### 2.5. B-Electronic Nose Detection

**Figure 1:**
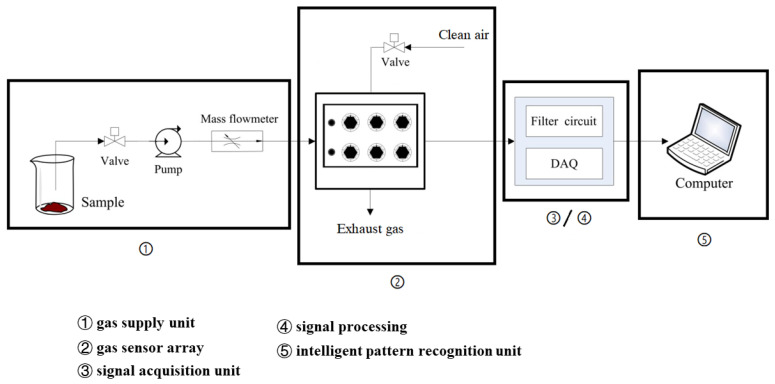
The schematic of the b-electronic nose system.

The volatile compound emissions in the glass bottle with beef samples that was sealed with the GPC film (2% chitosan concentration) or polyethylene film were analyzed by a self-made b-electronic nose system, as shown in Figure 1. When the olfactory cells of the biological olfactory organ get stimulation, it will transmit to the brain through the nerve and then express it back. In this work, inspired from the biological olfactory pattern, the b-electronic nose system is equipped with five units, including a gas supply unit, gas sensor array, signal acquisition unit, signal processing unit and intelligent pattern recognition unit. Six metal oxide gas sensors in the sensor array (manufactured by Figaro, Fukui, Japan and listed in the Table 1), which were named S1 to S6, were highly sensitive to volatile organic compound (VOC), ammonia, hydrogen sulfide, methane, carbon monoxide, carbon dioxide, alcohol, organic solvent, propane, butane and liquefied petroleum during food storage. The sensors were pre-heated for one hour before use.

The b-electronic nose system was placed in a clean room at 22 ± 1 ℃ and 60 ± 1% RH. Ground beef (5.0 g) was placed in a 50 mL glass bottle, sealed with the chitosan film or polyethylene film and then conditioned at 8 ℃ and 70% RH for different time periods prior to the determinations of the freshness of beef evaluated by electronic nose analysis. The sample’s headspace generation were incubated at 50 ℃ for 10 min. Headspace gas was pumped into the sensor chamber at a constant rate of 300 mL/min using an air pump. The acquisition frequency was 50 Hz and the acquisition time was 60 s. After the collection was completed, dry air was blown into the b-electronic nose system to clean it for 15 min before the next experiment was performed. Ten beef samples under the same storage condition were taken for subsequent analyses and each sample was analyzed three times.

In order to understand the b-electronic nose identification process and the repeatability of the beef samples sealed in two different films, the principal component analysis (PCA) method was used to analyze the multivariate data based on variable quantity restriction.

### 2.6. Statistical Analysis

The difference between factors and levels was evaluated by the analysis of variance (ANOVA). Duncan’s multiple range tests were used to compare the means to identify which groups were significantly different from other groups (*p* < 0.05). All data are presented as mean ± standard deviation

## 3. Results and Discussion

### 3.1. Fourier Transform Infrared (FT-IR) and X-Ray Diffraction (XRD) Analysis

**Figure 2:**
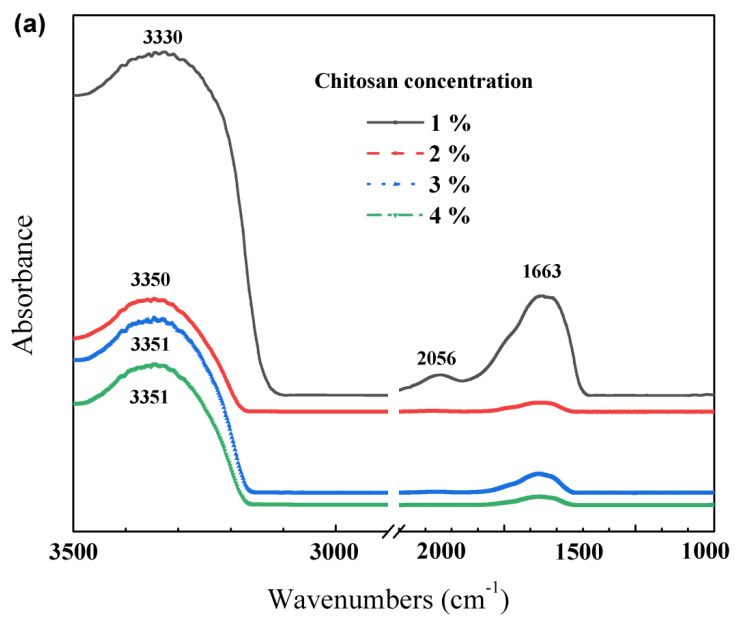

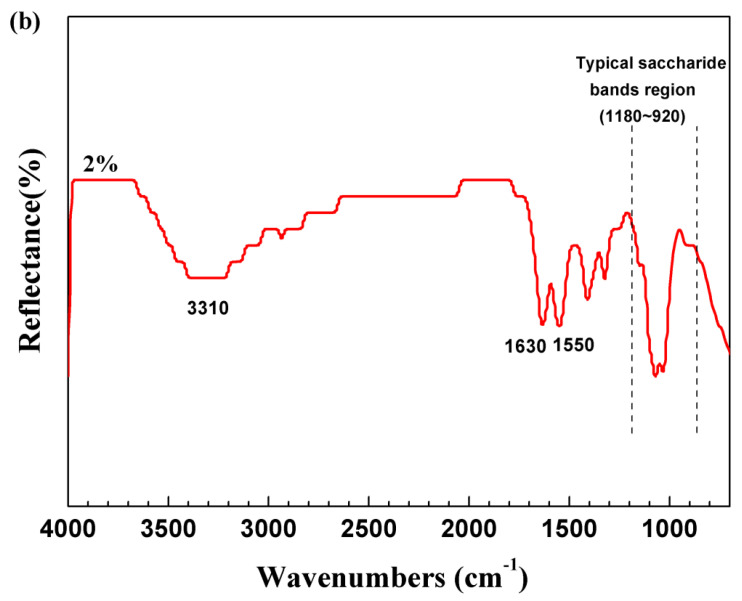
(**a**) FT-IR spectra for chitosan films and (**b**) ATR-FTIR spectra of the chitosan film with 2% chitosan concentration.

The FT-IR and ATR-FTIR spectra of the GPC films are shown in Figure 2. It can be seen from Figure 2a, in the FT-IR spectra of GPC films, that there was a broad band ranging from around 3500 to 3100 cm^−1^, which was attributed to the stretching vibration of N–H and hydrogen-bonded hydroxyl groups, and the peak located at 1663 cm^−1^ was associated with amide-I stretch. With the increasing of chitosan concentration, the intensities of the peaks located at 3330 and 1663 cm^−1^ of the chitosan films decreased, and the characteristic peak of chitosan films at 3330 cm^−1^ shifted to higher wavenumbers. These indicated that the formation of inter- and intra-molecular hydrogen bonding between chitosan and glycerol plasticizer and the chitosan concentration affected the polymer matrix structure and characteristics. The absorption band at wavenumber of 2056 cm^−1^ for the chitosan film containing 1% chitosan was a typical band associated with glycerol in addition to the contribution of the water absorption, according to the references [36,37]. The water contents of the chitosan films decreased with the increase in the chitosan concentration. When the chitosan concentration was 1%, the water content of the film was the lowest and the thickness was the thinnest, which favoured the test of FTIR, and the characteristic absorption peaks were easily presented. When the chitosan concentration was 2%, the intensities of the peaks located at 3350 and 1663 cm^−1^ of the chitosan film were the lowest, suggesting that the numbers of the polymer–polymer, polymer–plasticizer and polymer–solvent interactions were the largest and that there was not a phase separation, but was a good compatibility between the two main components of the chitosan films, which was consistent with the results that the mechanical properties increased noticeably at the chitosan concentration of 2%, presented in Section 3.4. ATR-FTIR is another powerful tool that can detect the possible interactions between the film components, but the transparency and color of the films were strictly required. Based on the results of color and opacity of the GPC films with different chitosan concentrations, the ATR-FTIR spectra of the GPC film with 2% chitosan concentration was expressed. As can be seen from Figure 2b, a broad band ranging from around 3600 to 3100 cm^−1^ was due to the stretching vibration of N–H and hydrogen-bonded hydroxyl groups. The peak at 1630 cm^−1^ was attributed to amide-I stretch and the peak at 1550 cm^−1^ was attributed to N–H bending, which were consistent with previous publications [35,38,39,40,41,42].

**Figure 3:**
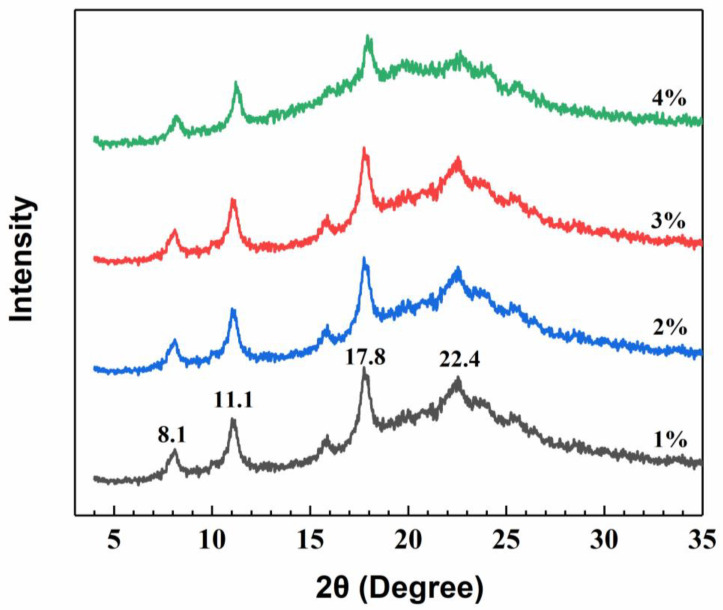
XRD patterns of chitosan films.

The crystalline structure of a packaging film plays an important role in maintaining its integrity during storage. XRD analysis was performed to check if the film preparation conditions affected the crystalline structure of films. Figure 3 showed the XRD patterns of GPC films with different chitosan concentrations. According to the study reported by Rhim et al. [43], the diffraction pattern of the chitosan power showed peaks at around 10.9° and 19.8°, which correspond to a hydrated crystalline structure due to the integration of water molecules in the crystal lattice and an amorphous structure of chitosan, respectively. As shown in Figure 3, the GPC films showed characteristic peaks at around 2θ = 8.1°, 11.1°, 17.8° and 22.4°, which were in agreement with the findings in previous publications [42,44,45]. The two peaks at 8.1° and 11.1° indicate a hydrated crystallite structure of chitosan, and the peak at 17.8° is attributed to the regular crystal lattice [36], while the broader peak at around 22.4° is due to an amorphous structure [44,45,46]. It is generally known that the structure of chitosan is strongly dependent on its origin, characteristics, such as degree of deacetylation and molecular weight, as well as processing treatment methods, for example dissolving, precipitation, heating and drying [43]. However, the crystalline structure of chitosan was not significantly affected through the chitosan film preparation process, as shown by the comparison of the XRD patterns in Figure 3.

### 3.2. Physicochemical Properties of the Chitosan Films

All chitosan solution was able to form homogeneous films that were flexible and free-standing, and the addition of glycerol resulted in smooth chitosan films that could be easily removed from the Petri dishes. It is worth noting that the GPC films had a slightly yellowish color, as well as that of the chitosan films prepared with 2.5% chitosan having low molecular weight or medium molecular weight [47]. The effects of chitosan concentration on the basic film properties, including film density, film solubility, swelling degree and color, are shown in Table 2. It can be noted that the film density increased along with the increasing of chitosan concentration. Water solubility is an important property of packaging films for their applications in food industries. When the packaging films are used to cover fruits and vegetables, films with low solubility will be required in order to maintain their structural integrity. When the packaging films, especially edible films, are used to package candies and cakes, high solubility of the films can be better [7,11,19,21]. It was found that although the GPC films in this work became rubbery when they were immersed in water, they still maintained their structural integrity. A substantial increase in the WS value of the GPC films was observed with the increase in chitosan concentration because chitosan is highly soluble in water, while the SD of the GPC films increased with increasing of chitosan concentration, and reached a maximum at 3% concentration, then declined at 4% concentration. SD is attributed to the network structure in GPC films, which was affected by the molecular weight and degree of deacetylation of chitosan, the hydrophilic properties of the glycerol and the preparation conditions of the films. When the chitosan concentration in the films was low, the increase in SD was due to the swelling property of the chitosan, while when the chitosan concentration increased to 4%, the content of glycerol in the chitosan films increased, which promoted the interactions with the polar groups, resulting in less polar groups being accessible to interact with water molecules, therefore the GPC films with 4% chitosan concentration had the lowest SD.

The color of packaging films is of primary importance, which determines the overall acceptability of consumers because consumers want to observe the visual characteristics of the product in the packaging materials. Table 2 showed the results of color measurements for the GPC films. It can be seen that the luminosity (L*) value of chitosan film decreased significantly with the increase in chitosan concentration; in other words, the chitosan films became darker when the chitosan concentration was higher. When parameter a* has negative values, it means that the green color is present in lesser or greater intensity at the GPC films with 1% and 2% chitosan concentration. The a* and b* values of the chitosan films significantly increased with the increasing of chitosan concentration, indicating that the chitosan films were redder and yellower. Total color differences (ΔE*) and color intensity (C*) could represent the color difference among all the chitosan films and significantly increased with the chitosan concentration increasing, as shown in Table 2. The higher ΔE* gave rise to more colored packaging films, which was expected since the changes in color were attributed to the concentration of chitosan. A similar trend was also reported by Ren et al. [35] working on chitosan and corn starch blend films with different chitosan concentrations.

### 3.3. Water Vapor Permeability (WVP)

**Figure 4:**
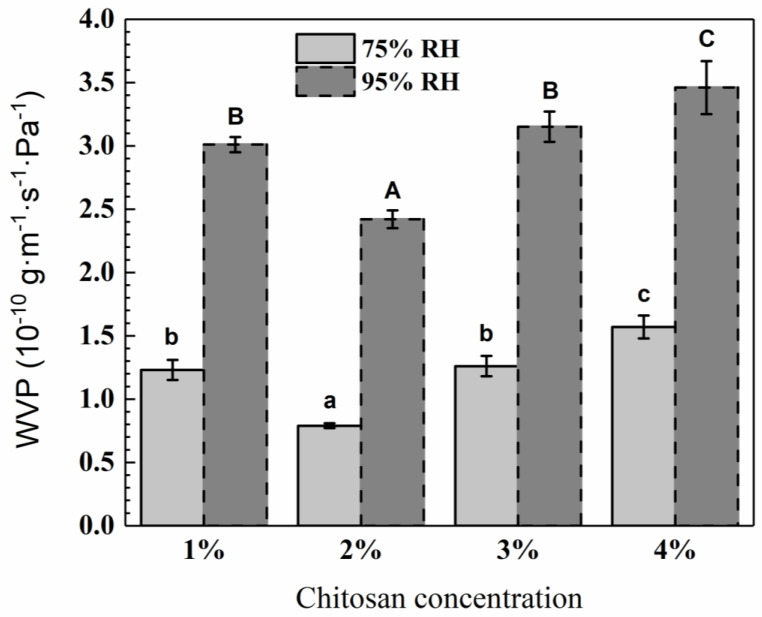
WVP of chitosan films. Small letters in 75% RH group, *p*  <  0.05; capital letters in 95% RH group, *p*  <  0.05.

WVP is a proportionality constant that is assumed to be independent of the water vapor pressure gradient applied across the packaging films. The effects of chitosan concentration on the WVP of the GPC films are presented in Figure 4. The GPC films showed WVP values ranging from 0.79 × 10^−10^ g·m^−1^s^−1^Pa^−1^ to 1.57 × 10^−10^ g·m^−1^s^−1^Pa^−1^ under 75% RH, and WVP values ranging from 2.42 × 10^−10^ g·m^−1^s^−1^Pa^−1^ to 3.46 × 10^−10^ g·m^−1^s^−1^Pa^−1^ under 95% RH. GPC films in this work exhibited WVP values within the range of those reported for chitosan films obtained by dissolving 2.5% chitosan with different chitosan molecular weights in 1.25% acetic acid [47]. The WVP values of chitosan films were 4.14 × 10^−10^ g·m^−1^s^−1^Pa^−1^, 3.38 × 10^−10^ g·m^−1^s^−1^Pa^−1^ and 4.55 × 10^−11^ g·m^−1^s^−1^Pa^−1^ for chitosan of low molecular weight, medium molecular weight and high molecular weight, respectively [47].

The permeability of GPC film with 1% chitosan concentration under 75% RH (1.23 × 10^−10^ g·m^−1^s^−1^Pa^−1^) in this work was higher than the chitosan film plasticized without glycerol (0.45 × 10^−10^ g·m^−1^s^−1^Pa^−1^) reported by Garcia et al. [48] who prepared chitosan film by solubilizing 1% (*w*/*w*) chitosan into 1% (*v*/*v*) aqueous acetic acid solution. The permeability of GPC film with 4% chitosan concentration under 95% RH (3.46 × 10^−10^ g·m^−1^s^−1^Pa^−1^) was higher than the chitosan film with 4 wt% chitosan concentration plasticized with glycerol at 30 wt% of the chitosan powder (11.92 g·mm m^-2^ day^−1^ kPa^−1^ = 1.38 × 10^−10^ g·m^−1^s^−1^Pa^−1^) [49]. The higher WVP of GPC films in this study may be due to the addition of hydrophilic glycerol and the larger amount of hydroxyl and amino groups. On the one hand, free hydroxyl and amino groups would improve the interactions with water, which would favor the water vapor transmission through the chitosan films. On the other hand, glycerol as the plasticizer could make the distance between the molecules larger and decrease the number of the intra-molecular hydrogen, making the chitosan network become sparser and be easy to adsorb and desorb water molecules [50].

At 75% RH and 95% RH, the WVP of the GPC films decreased with the increasing of the chitosan concentration, and reached a minimum at 2% concentration, then increased at higher chitosan concentrations. The factors that could affect the WVP of GPC films include the chitosan molecular weight, degree of deacetylation of chitosan, the type and additive amounts of plasticizers and the preparation method of the films, which would influence the network structure in chitosan films [49]. The largest numbers of hydroxyl groups and glycerol would result in the highest WVP, while the GPC film with 2% chitosan concentration had the lowest WVP. The reason was that when the chitosan concentration was 2%, the interactions between chitosan and glycerol were developed and lots of hydroxyl and amino groups of chitosan were used for the hydrogen bond formation, which reduced the hydrophilic groups’ availability of chitosan to the greatest extent; therefore, it was not easy for water vapor to transmit through the chitosan films, while the WVP of GPC films increased with the increase in chitosan concentration due to the more hydrophilic groups (hydroxyl and amino groups) left. A good biodegradable or edible packaging film must have, as a major function, the hindering of water vapor transfer between food and the surrounding atmosphere, or between two components of heterogeneous food products, which requires the WVP value of these films to be as low as possible. Having a minimum of WVP for the GPC film with 2% chitosan concentration is of great importance in evaluating the chitosan films for their use in food packaging, protective coatings and other applications where barrier efficiency is needed.

### 3.4. Mechanical Properties

**Figure 5:**
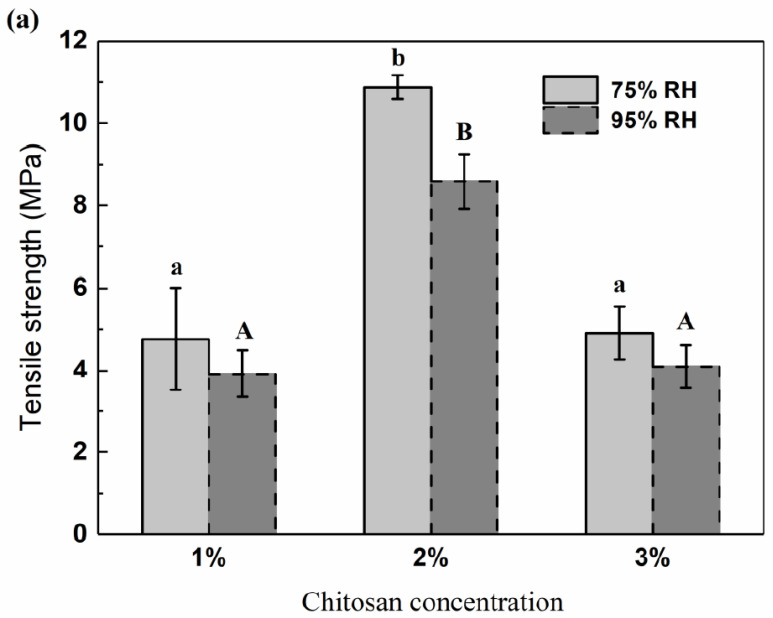

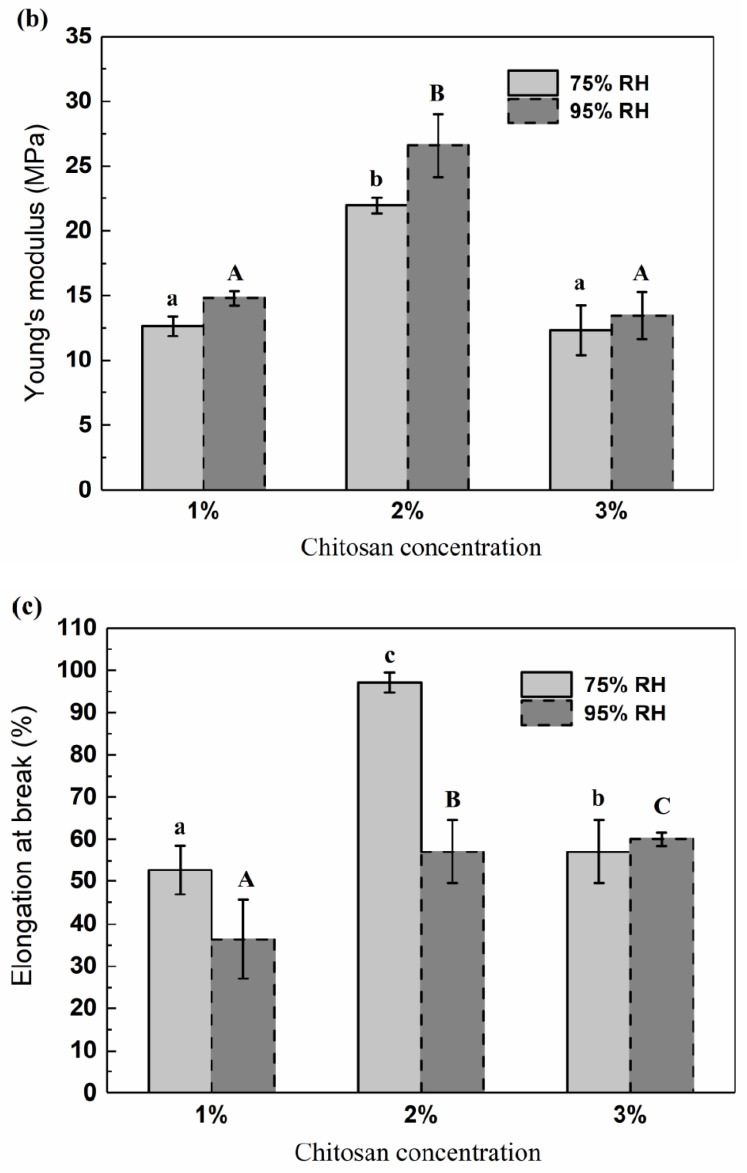
Mechanical properties of chitosan films: (**a**) tensile strength, (**b**) Young’s modulus and (**c**) elongation at break. Small letters in 75% RH group, *p*  <  0.05; capital letters in 95% RH group, *p*  <  0.05.

The production of packaging materials requires them to be resistant to breakage and abrasion in order to protect the packaged products during handling and transport, and, at the same time, maintaining their flexibility to adapt to eventual deformations of the products. Therefore, a certain amount of mechanical strength and extensibility is needed for biodegradable or edible packaging films in order to maintain their integrity and extending ability as materials for food and pharmaceutical packaging applications. The mechanical properties, including the tensile strength, Young’s modulus and elongation at break, of the GPC films after equilibrium at 75% RH and 95% RH are shown in Figure 5.

In general, plasticized chitosan films exhibited the stress–strain behavior of ductile polymeric materials. The stress–strain curves of GPC films with different chitosan concentrations showed the typical pattern of extremely deformable and flexible materials, because they exhibited low tensile strength and Young’s modulus and high elongation at break (Figure 5). These values were within the range of those previously reported by other authors for chitosan films [43,47,48,51,52], and differences may be attributed to chitosan film composition and material suppliers, film preparation methods, as well as storage conditions.

**Figure 6:**
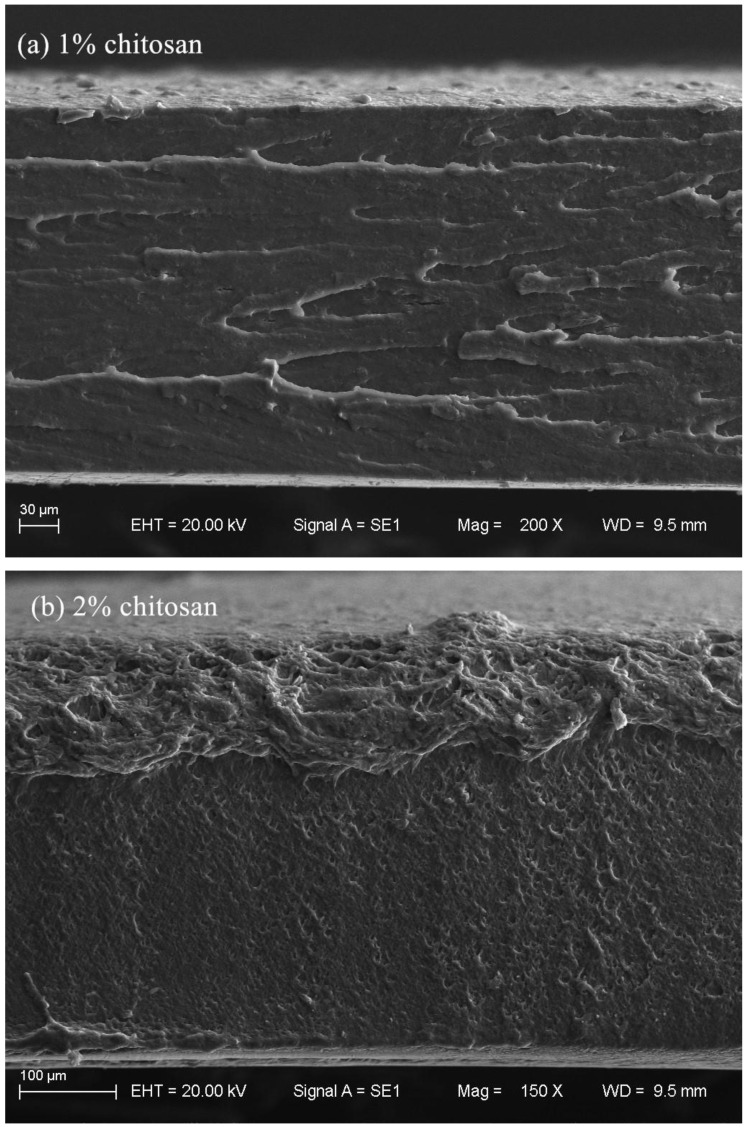

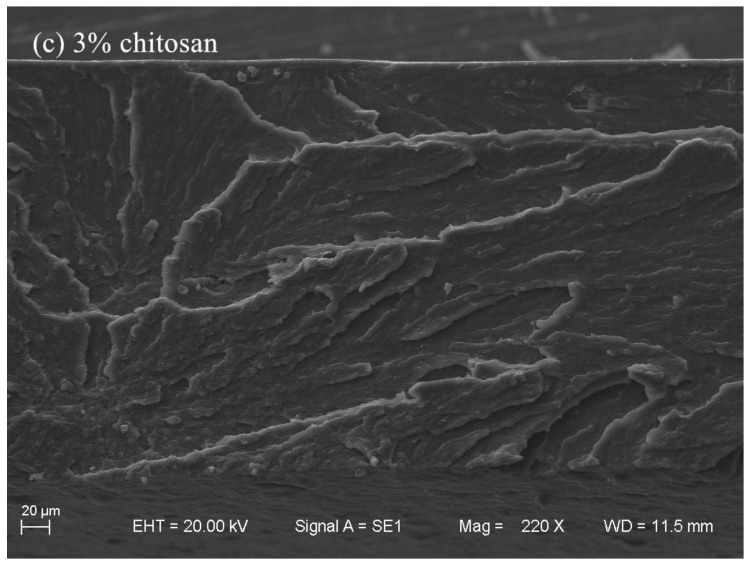
SEM micrographs of the tensile fractured surfaces’ panorama of chitosan films. Chitosan concentration is (**a**) 1%, (**b**) 2% and (**c**) 3%

According to Figure 5, in the case of the GPC films, the film with 1% chitosan concentration exhibited a tensile strength closer to that of the one with 3% chitosan concentration, meanwhile, the chitosan film with 2% chitosan concentration showed the highest value under both 75% RH and 95% RH, which can be also explained by the results of SEM analysis (Figure 6). It was observed that although GPC films exhibited rough tensile fractured surfaces, the cross section of chitosan film with 2% chitosan concentration showed a more continuous surface and a more compact structure without separation of phases between the chitosan and glycerol (Figure 6), suggesting that 2% chitosan and the glycerol plasticizer during the film preparation process were highly compatible. Similar trends for elongation at break at 75% RH and Young’s modulus were observed in the chitosan films with different chitosan concentrations, while elongation at break of the GPC films at 95% RH increased with the increasing of the chitosan concentration. This could be attributed to the effects of the chitosan concentration on the type and number of polymer–polymer, polymer–plasticizer and polymer–solvent interactions, which determined the polymer matrix structure and characteristics. The increased flexibility of the GPC films at higher chitosan concentrations could be due to the interaction of plasticizer–polymer chains, which facilitated the sliding of the chain and thus helped to improve the overall flexibility and chain mobility [35,53]. An explanation was argued by Bof et al. [47], who stressed that the presence of the plasticizer in the chitosan films made polymer–plasticizer and polymer–solvent interactions become more important and the development of high extensible materials was favored. Martínez-Camacho et al. [53], working on chitosan-based films, also stressed that the plasticizer in the films reduced the necessary effort for the deformation, as well as the deformation of the films before their rupture. In addition, with the increasing of chitosan concentration, the number of NH^3+^ groups of chitosan in the film-forming solution increased, and when the concentration exceeded a critical value, it was very difficult to form homogeneous interactions, resulting in the poorer mechanical properties of the GPC films.

The film with 4% chitosan concentration is not shown in Figure 5 and Figure 6. This is because that the chitosan films became darker with the increase in the chitosan concentration, and the film with 4% chitosan possessed the worst physicochemical and moisture barrier properties, which makes the film with 4% chitosan concentration unsuitable for packaging film. Furthermore, after the specimens, 50 mm long dumbbells with 4 mm neck width, were cut from the film and balanced in 75% or 95% RH, there would be cracks on the surface of the specimens, which made the test data inaccurate.

### 3.5. Sensory Evaluation

**Figure 7:**
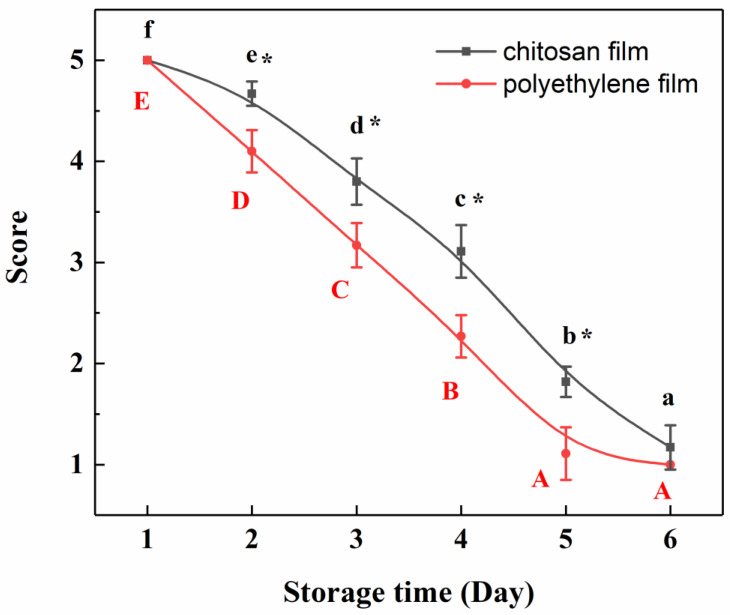
Sensory evaluation results of ground beef sealed with chitosan film and polyethylene film. Small letters in the chitosan film group, *p*  <  0.05; capital letters in the polyethylene film group, *p*  <  0.05; *, *p*  <  0.05, compared with sensory scores of beef samples sealed with polyethylene film.

Since the film with 2% chitosan possessed the best physicochemical, moisture barrier and mechanical properties, which were closer to those of plant epidermis, only the chitosan film with 2% chitosan concentration was used to seal the beef sample for determining the beef’s freshness, which was evaluated by sensory evaluation and b-electronic nose analysis. The color, odor, viscosity and resilience of the beef samples conditioned at 8 °C and 70% RH for 1 to 6 days were evaluated and the results are shown in Figure 7. It can be seen that, initially, the beef freshness was excellent and the scores decreased significantly (*p* < 0.05) with the increase in storage time; in other words, longer storage time led to the poorer freshness of the ground beef. Similar trends were also reported in the literature working on pork [54], yellow chicken meat [55] and smoked bacon [56]. For the beef sample stored for 1 to 2 days, the freshness characteristics scored above four, which corresponded to the fresh group. For the beef sample stored for 3 to 4 days, the scores were lower than four and equal to or greater than two, corresponding to sub-fresh group, while the scores of 5–6 days’ storage samples were lower than two and the beef deteriorated and even rotted. In addition, the freshness of the beef sealed with chitosan film was better than that with polyethylene film. These results of the freshness suggested that the chitosan film could keep beef samples fresher in a certain period of storage time and delay the deterioration of the beef, which is supported by the antimicrobial tests (not shown in this study), where chitosan film has a more powerful antimicrobial activity against Gram-negative bacteria *Escherichia coli* (*E. coli*) and Gram-positive bacteria *Staphylococcus aureus* (*S. aureus*) than polyethylene film. Moreover, the scoring test, as a very simple and fast sensory evaluation method, could effectively distinguish beef samples with different storage times and be used as a reference for the electronic nose’s discriminant factor analysis. The sensory fresh and sub-fresh groups were then used as the subjects for the b-electronic nose analysis.

### 3.6. B-Electronic Nose Analysis

**Figure 8:**
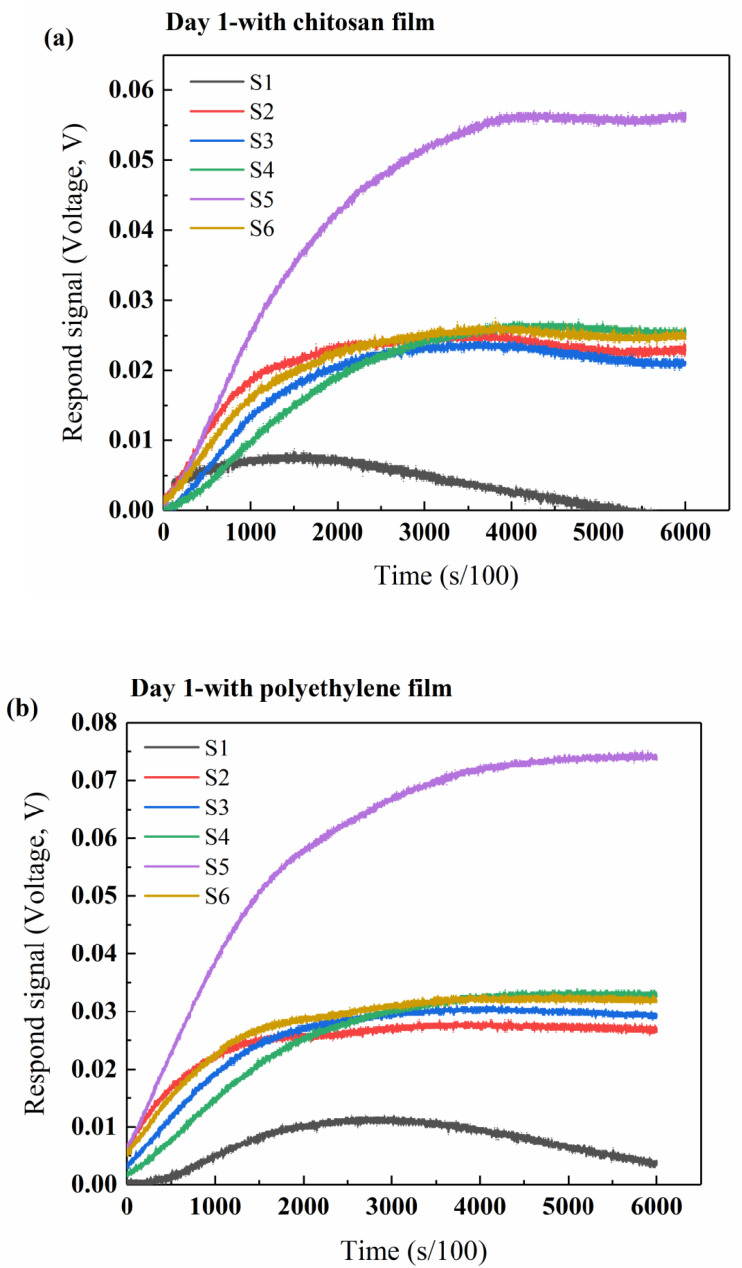

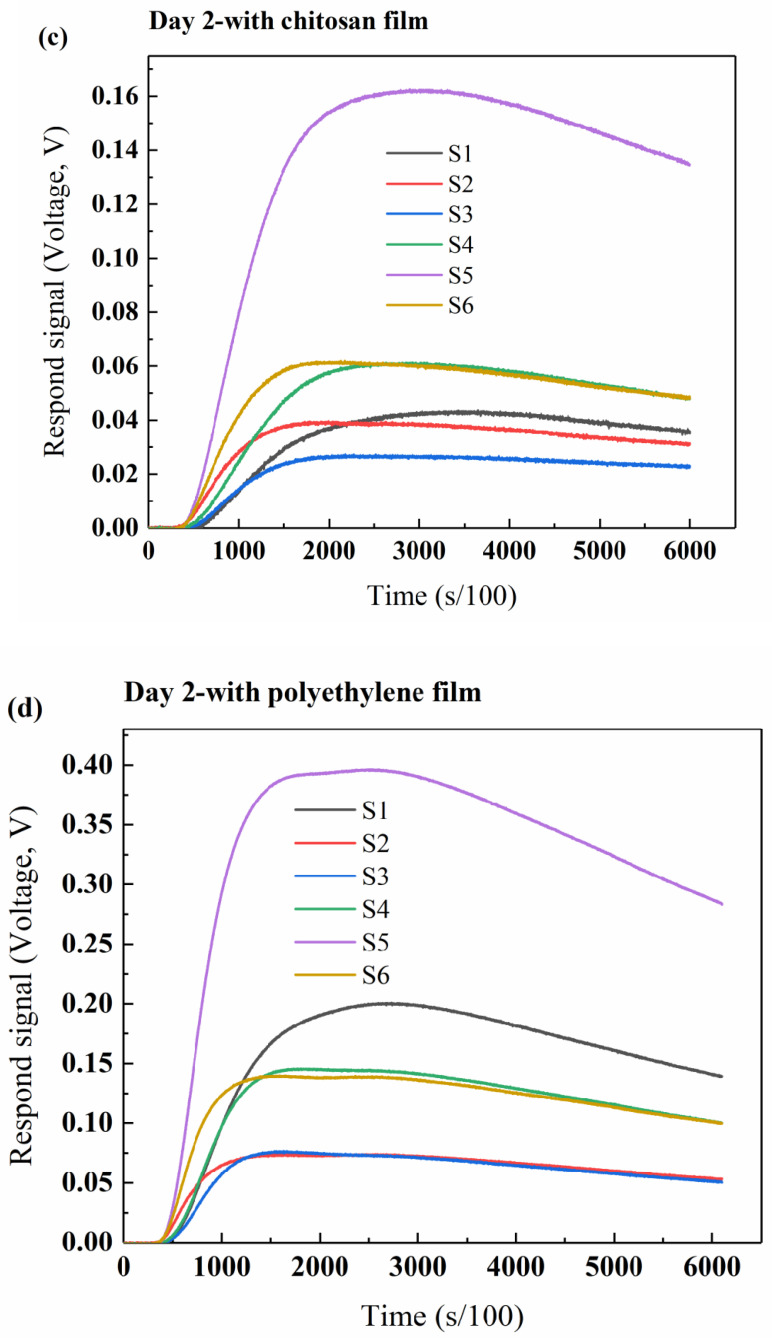

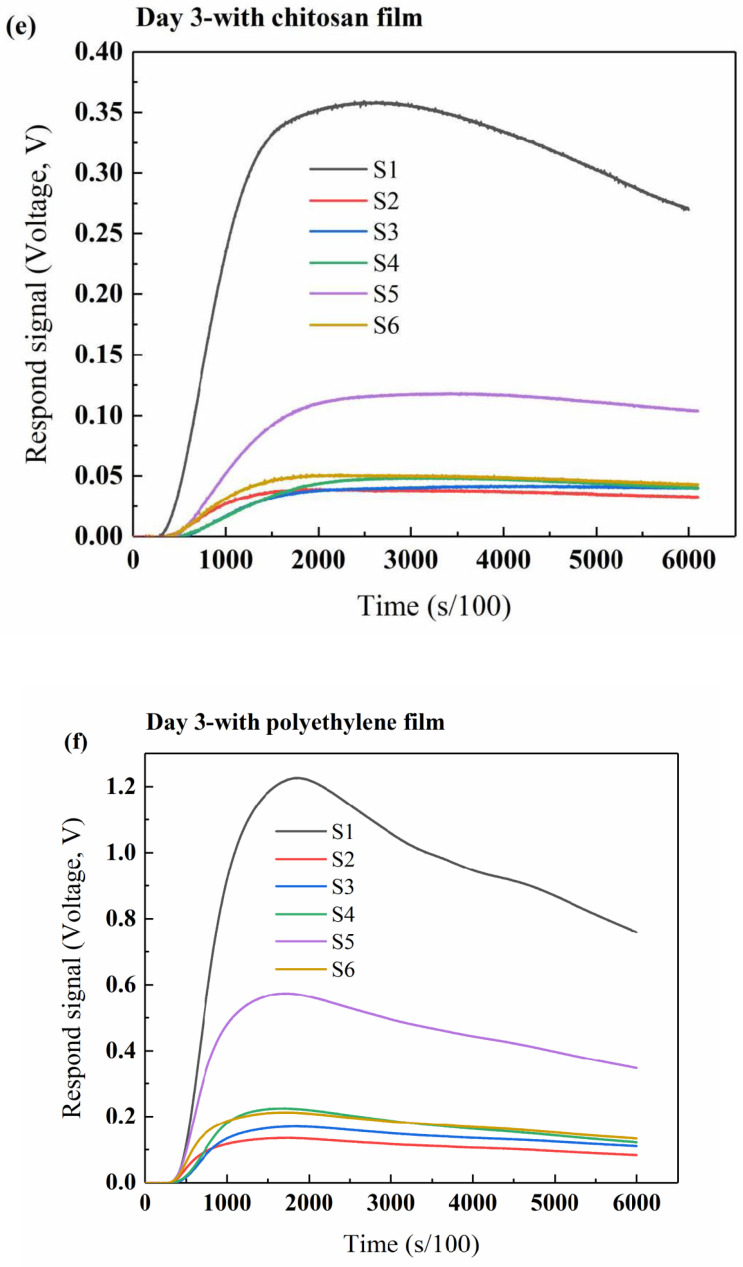
The typical original electronic nose response signals of ground beef sealed with chitosan film and polyethylene film at 8 °C for different amounts of days. The typical original electronic nose response signals of ground beef sealed with chitosan film for (**a**) 1 day, (**c**) 2 day and (**e**) 3 day, and polyethylene film for (**b**) 1 day, (**d**) 2 day and (**f**) 3 day at 8 °C.

The b-electronic nose is always designed to detect and distinguish between complex odors according to the sensitivity ranges of the gas sensors [30,32,40,56,57]. Therefore, in food packaging, industry properties, such as shelf-life and freshness, can be investigated for any food or materials that give out volatile compounds. In this study, the b-electronic nose was used to monitor changes in the beef’s volatile compounds sealed with the chitosan films or polyethylene film during storage. The typical original electronic nose response signals of beef stored for different amounts of days are shown in Figure 8, where each line of different colors in the figure represents one gas sensor. Due to the continuous accumulation and reaction of volatile gases on the surface of the gas sensors, basically, for all the gas sensors, the response intensity increased from zero and the increase in strength was quick in the first 10 to 20 s detection; afterwards, the curves reached a maximum value and gradually became level. The different responses of the sensors indicated that the composition and content of volatile gases in the beef samples sealed with different films and stored for different amounts of days are different. During the first 2 days, the final response intensity of S5 (TGS2620) for the beef samples sealed with chitosan film or polyethylene film was the highest among all sensors (Figure 8a-d), revealing that alcohol is the main volatile component in the early days of beef storage.

At 1-day of storage of the beef sample, the final response intensity of S1 (TGS2602), which can sense VOC, ammonia and hydrogen sulfide, was the lowest, and there were no significant differences among those of S2–4 and S6, whose values were between those of S5 and S1 (Figure 8a,b), while the S1 sensor gave a response that increased with the increasing of the storage time and became the biggest contributor at 3-days’ storage (Figure 8e,f), which was probably due to the partial correspondence between the sensor’s sensitivity and the compounds reported as major volatiles in beef’s headspace generation.

In addition, sensors tended to respond more to the beef samples sealed with polyethylene film than those with chitosan film at the same storage time. In other words, the intensity of the corresponding sensor response for the beef sample sealed with polyethylene film was stronger than that for the sample sealed with chitosan film conditioned at the same time. The electronic nose data confirmed that there was an effect of the packaging film on the properties of the beef samples. The sensors S5 and S1 had stronger responses during the storage of beef samples, so the maximum value of these two sensors was selected as the characteristic variable for PCA analysis. The contribution rate of two principal components of the beef samples was 92.17%, which indicated that two principal components could provide enough information to explain the difference in the headspace generation of beef samples emitted during different storage times. Using the sensory evaluation results as reference, the principal component analysis showed that the b-electronic nose could effectively distinguish and judge the freshness of beef samples, suggesting that the b-electronic nose is promising for the detection applications of beef’s freshness.

## 4. Conclusions

Glycerol plasticized chitosan (GPC) films were successfully prepared by solution casting. The effect of chitosan concentration (1, 2, 3 and 4%) on physicochemical, mechanical and water vapor barrier properties of the GPC films, and the effectiveness of traditional sensory evaluation and b-electronic nose methods in investigating the freshness of ground beef sealed with chitosan film or polyethylene film, were evaluated. Characterization results suggested that the hydrogen bonding between chitosan and plasticizer formed as confirmed from the shift of the main peaks to higher wavenumbers and the decrease in characteristic peak intensities in FTIR. A substantial increase (*p* < 0.05) in the WS value of GPC films was observed with the increase in chitosan concentration, while the SD of the GPC films increased with the increasing of the chitosan concentration and then declined at higher concentrations. The color results showed that the GPC films became redder and yellower with the increasing of the chitosan concentration. The WVP of the GPC films at 75% RH and 95% RH decreased with the increasing of the chitosan concentration, and reached a minimum at 2% concentration, then increased at higher chitosan concentrations. The GPC film with 2% chitosan concentration showed the highest tensile strength, Young’s modulus and elongation at break values. Sensory evaluation (scoring test), a very simple and fast method, could be used as a reference for the electronic nose detection. The principal component analysis showed that b-electronic nose perfectly confirmed findings from sensory tests and could distinguish and judge the freshness of beef samples stored for different lengths of time. These should provide clear pictures on the real potential use of GPC film as an active packaging material and of b-electronic nose as a promising detection method for the shelf-life and quality of meat products.

## Figures and Tables

**Table 1:** Gas sensor array and its properties.

Sensor Number	Series Name	Sensitive Substances
S1	TGS2602	VOC, ammonia, hydrogen sulfide
S2	TGS2611	methane
S3	TGS2442	carbon monoxide
S4	TGS4161	carbon dioxide
S5	TGS2620	alcohol, other organic solvent
S6	TGS2610	propane, butane, liquefied petroleum

**Table 2:** Density, swelling degree (SD) and solubility (WS) in water and color of the chitosan films.

Chitosan Concentration	Density (g cm^−3^)	SD (%)	WS (%)	Color				
				L*	a*	b*	ΔE*	C*
1 %	0.87 ± 0.01a	85.93 ± 6.72b	30.04 ± 0.10a	89.61 ± 0.34a	−1.73 ± 0.06a	14.73 ± 1.28a	14.91 ± 1.26a	4.99 ± 0.34a
2 %	0.96 ± 0.01b	92.76 ± 7.13c	32.57 ± 0.16b	86.93 ± 0.22a	−2.90 ± 0.04b	20.93 ± 0.90b	21.72 ± 0.91b	7.86 ± 0.22b
3 %	1.24 ± 0.02d	146.71 ± 9.25d	32.91 ± 0.12b	67.82 ± 0.38b	15.66 ± 0.51c	79.11 ± 0.39d	84.48 ± 0.48c	31.40 ± 0.51c
4 %	1.16 ± 0.01c	79.94 ± 5.69a	33.01 ± 0.11c	50.88 ± 3.30c	33.33 ± 2.36d	64.58 ± 7.41c	84.79 ± 5.61c	55.44 ± 4.04d

Different letters in the same column indicate significantly different (*p* < 0.05) when analyzed by Duncan’s New Multiple Range Test.

## References

[1] Mahieu A., Terrié C., Youssef B. (2015). Thermoplastic starch films and thermoplastic starch/polycaprolactone blends with oxygen-scavenging properties: Influence of water content. Ind. Crop. Prod..

[2] Suppakul P., Miltz J., Sonneveld K., Bigger S. (2003). Active Packaging Technologies with an Emphasis on Antimicrobial Packaging and its Applications. J. Food Sci..

[3] Appendini P., Hotchkiss J.H. (2002). Review of antimicrobial food packaging. Innov. Food Sci. Emerg. Technol..

[4] Dang K.M., Yoksan R. (2016). Morphological characteristics and barrier properties of thermoplasticstarch/chitosan blown film. Carbohydr. Polym..

[5] Abdollahi M., Rezaei M., Farzi G. (2012). A novel active bionanocomposite film incorporating rosemary essential oil and nanoclay into chitosan. J. Food Eng..

[6] Muzzarelli R.A.A., Boudrant J., Meyer D., Manno N., DeMarchis M., Paoletti M.G. (2012). A tribute to Henri Braconnot, precursor of the carbohydrate polymersscience on the chitin bicentennial. Carbohydr. Polym..

[7] Elsabee M.Z., Abdou E.S. (2013). Chitosan based edible films and coatings: A review. Mater. Sci. Eng. C.

[8] Aider M. (2010). Chitosan application for active bio-based films production and potential in the food industry: Review. LWT Food Sci. Technol..

[9] Bonilla J., Atarés L., Vargas M., Chiralt A. (2013). Properties of wheat starch film-forming dispersions and films as affected by chitosan addition. J. Food Eng..

[10] Arfat Y.A., Benjakul S., Vongkamjan K., Sumpavapol P., Yarnpakdee S. (2015). Shelf-life extension of refrigerated sea bass slices wrapped with fish protein isolate/fish skin gelatin-ZnO nanocomposite film incorporated with basil leaf essential oil. J. Food Sci. Technol..

[11] Withouck H., Boeykens A., Broucke M.V., Moreira M.M., Delerue-Matos C., De Cooman L. (2019). Evaluation of the impact of pre-treatment and extraction conditions on the polyphenolic profile and antioxidant activity of Belgium apple wood. Eur. Food Res. Technol..

[12] Markowicz J., Uram Ł., Sobich J., Mangiardi L., Maj P., Rode W. (2019). Antitumor and anti-nematode activities of α-mangostin. Eur. J. Pharmacol..

[13] Dutta P., Tripathi S., Mehrotra G., Dutta J. (2009). Perspectives for chitosan based antimicrobial films in food applications. Food Chem..

[14] Darmadji P., Izumimoto M. (1994). Effect of chitosan in meat preservation. Meat Sci..

[15] Ouattara B., Simard R., Piette J.-P.G., Bégin A., Holley R. (2000). Diffusion of Acetic and Propionic Acids from Chitosan-based Antimicrobial Packaging Films. J. Food Sci..

[16] Artharn A., Prodpran T., Benjakul S. (2009). Round scad protein-based film: Storage stability and its effectiveness for shelf-life extension of dried fish powder. LWT Food Sci. Technol..

[17] Di Pierro P., Chico B., Villalonga R., Mariniello L., Damiao A.E., Masi P., Porta R. (2006). Chitosan−Whey Protein Edible Films Produced in the Absence or Presence of Transglutaminase: Analysis of Their Mechanical and Barrier Properties. Biomacromolecules.

[18] Kołodziejska I., Piotrowska B. (2007). The water vapour permeability, mechanical properties and solubility of fish gelatin–chitosan films modified with transglutaminase or 1-ethyl-3-(3-dimethylaminopropyl) carbodiimide (EDC) and plasticized with glycerol. Food Chem..

[19] Vásconez M.B., Flores S.K., Campos C.A., Alvarado J., Gerschenson L.N. (2009). Antimicrobial activity and physical properties of chitosan–tapioca starch based edible films and coatings. Food Res. Int..

[20] De Abreu D.P., Losada P.P., Angulo I., Cruz J. (2007). Development of new polyolefin films with nanoclays for application in food packaging. Eur. Polym. J..

[21] De Moura M.R., Aouada F.A., Avena-Bustillos R.J., McHugh T., Krochta J., Mattoso L.H.C., Aouada F.A. (2009). Improved barrier and mechanical properties of novel hydroxypropyl methylcellulose edible films with chitosan/tripolyphosphate nanoparticles. J. Food Eng..

[22] Chillo S., Flores S., Mastromatteo M., Conte A., Gerschenson L., Del Nobile M.A. (2008). Influence of glycerol and chitosan on tapioca starch-based edible film properties. J. Food Eng..

[23] Ren L., Fu Y., Chang Y., Jiang M., Tong J., Zhou J. (2016). Performance improvement of starch films reinforced with starch nanocrystals (SNCs) modified by cross-linking. Starch Stärke.

[24] Vieira M.G.A., Da Silva M.A., Dos Santos L.O., Beppu M.M. (2011). Natural-based plasticizers and biopolymer films: A review. Eur. Polym. J..

[25] Sessini V., Arrieta M., Fernández-Torres A., Peponi L. (2018). Humidity-activated shape memory effect on plasticized starch-based biomaterials. Carbohydr. Polym..

[26] Cervera M.F., Karjalainen M., Airaksinen S., Rantanen J., Krogars K., Heinämäki J., Colarte A.I., Yliruusi J. (2004). Physical stability and moisture sorption of aqueous chitosan–amylose starch films plasticized with polyols. Eur. J. Pharm. Biopharm..

[27] Arrieta M.P., Peltzer M.A., Garrigós M.D.C., Jiménez A. (2013). Structure and mechanical properties of sodium and calcium caseinate edible active films with carvacrol. J. Food Eng..

[28] Priyadarshi R., Sauraj, Kumar B., Negi Y.S. (2018). Chitosan film incorporated with citric acid and glycerol as an active packaging material for extension of green chilli shelf life. Carbohydr. Polym..

[29] Reddy N., Yang Y. (2010). Citric acid cross-linking of starch films. Food Chem..

[30] Wojnowski W., Kalinowska K., Majchrzak T., Płotka-Wasylka J., Namieśnik J. (2019). Prediction of the Biogenic Amines Index of Poultry Meat Using an Electronic Nose. Sensors.

[31] Torri L., Piochi M. (2016). Sensory methods and electronic nose as innovative tools for the evaluation of the aroma transfer properties of food plastic bags. Food Res. Int..

[32] Wang Q., Li L., Ding W., Zhang D.Q., Wang J., Reed K., Zhang B. (2019). Adulterant identification in mutton by electronic nose and gas chromatography-mass spectrometer. Food Control..

[33] Zamudio-Flores P.B., Torres A.V., Salgado-Delgado R., Bello-Pérez L.A. (2010). Influence of the oxidation and acetylation of banana starch on the mechanical and water barrier properties of modified starch and modified starch/chitosan blend films. J. Appl. Polym. Sci..

[34] Mayachiew P., Devahastin S. (2010). Effects of drying methods and conditions on release characteristics of edible chitosan films enriched with Indian gooseberry extract. Food Chem..

[35] Ren L.L., Yan X.X., Zhou J., Su X.G. (2017). Influence of chitosan concentration on mechanical and barrierproperties of corn starch/chitosan films. Int. J. Biol. Macromol..

[36] Souza R.C.R., Andrade C.T. (2002). Investigation of the gelatinization and extrusion processes of corn starch. Adv. Polym. Technol..

[37] Liu H., Adhikari R., Guo Q., Adhikari B. (2013). Preparation and characterization of glycerol plasticized (high-amylose) starch–chitosan films. J. Food Eng..

[38] Mathew S., Brahmakumar M., Abraham T.E. (2006). Microstructural imaging and characterization of the mechanical, chemical, thermal, and swelling properties of starch–chitosan blend films. Biopolymers.

[39] Yusof Y.M., Shukur M.F., Illias H.A., Kadir M.F.Z. (2014). Conductivity and electrical properties of corn starch–chitosan blend biopolymer electrolyte incorporated with ammonium iodide. Phys. Scr..

[40] Bourtoom T., Chinnan M. (2008). Preparation and properties of rice starch–chitosan blend biodegradable film. LWT Food Sci. Technol..

[41] Park P.-J., Je J.-Y., Kim S.-K. (2004). Free radical scavenging activities of differently deacetylated chitosans using an ESR spectrometer. Carbohydr. Polym..

[42] Shukur M.F., Yusof Y.M., Zawawi S.M.M., Illias H.A., Kadir M.F.Z. (2013). Conductivity and transport studies of plasticized chitosan-based proton conducting biopolymer electrolytes. Phys. Scr..

[43] Rhim J.-W., Hong S.-I., Park H.-M., Ng P.K.W. (2006). Preparation and Characterization of Chitosan-Based Nanocomposite Films with Antimicrobial Activity. J. Agric. Food Chem..

[44] Zhai M., Zhao L., Yoshii F., Kume T. (2004). Study on antibacterial starch/chitosan blend film formed under the action of irradiation. Carbohydr. Polym..

[45] Jiménez A., Fabra M.J., Talens P., Chiralt A. (2012). Effect of sodium caseinate on properties and ageing behaviour of corn starch based films. Food Hydrocoll..

[46] Giannakas A., Grigoriadi K., Leontiou A., Barkoula N.-M., Ladavos A. (2014). Preparation, characterization, mechanical and barrier properties investigation of chitosan–clay nanocomposites. Carbohydr. Polym..

[47] Bof M.J., Bordagaray V.C., Locaso D.E., García M.A. (2015). Chitosan molecular weight effect on starch-composite film properties. Food Hydrocoll..

[48] García M.A., Pinotti A., Zaritzky N. (2006). Physicochemical, Water Vapor Barrier and Mechanical Properties of Corn Starch and Chitosan Composite Films. Starch Stärke.

[49] Wang L., Dong Y., Men H., Tong J., Zhou J. (2013). Preparation and characterization of active films based on chitosan incorporated tea polyphenols. Food Hydrocoll..

[50] Mei J., Yuan Y., Wu Y., Li Y. (2013). Characterization of edible starch–chitosan film and its application in the storage of Mongolian cheese. Int. J. Biol. Macromol..

[51] Pelissari F.M., Grossmann M.V., Yamashita F., Pineda E.A. (2009). Antimicrobial, mechanical, and barrier properties of cassava starch-chitosanfilms incorporated with oregano essential oil. J. Agric. Food Chem..

[52] Zhong Y., Li Y., Zhao Y. (2012). Physicochemical, Microstructural, and Antibacterial Properties of ??Chitosan and Kudzu Starch Composite Films. J. Food Sci..

[53] Martínez-Camacho A., Cortez-Rocha M.O., Ezquerra-Brauer J., Graciano-Verdugo A., Rodriguez-Félix F., Castillo-Ortega M.M., Yépiz-Gómez M., Plascencia-Jatomea M. (2010). Chitosan composite films: Thermal, structural, mechanical and antifungal properties. Carbohydr. Polym..

[54] Chen J., Gu J., Zhang R., Mao Y., Tian S. (2019). Freshness Evaluation of Three Kinds of Meats Based on the Electronic Nose. Sensors.

[55] Lu W., Wu Y., Guo Q., Ren L., Zhu P., Xu L., Chang G., Chen G. (2017). Establishment of a Freshness-Evaluating Standard for Chilled Yellow Chicken Meat. Food Anal. Methods.

[56] Li X., Zhu J., Li C., Ye H., Wang Z., Wu X., Xu B. (2018). Evolution of Volatile Compounds and Spoilage Bacteria in Smoked Bacon during Refrigeration Using an E-Nose and GC-MS Combined with Partial Least Squares Regression. Molecules.

[57] Chang Z., Sun Y., Zhang Y., Gao Y., Weng X., Chen D., David L., Xie J. (2018). Bionic Optimization Design of Electronic Nose Chamber for Oil and Gas Detection. J. Bionic Eng..

